# Clinically observed deletions in SARS‐CoV‐2 Nsp1 affect its stability and ability to inhibit translation

**DOI:** 10.1002/1873-3468.14354

**Published:** 2022-04-25

**Authors:** Pravin Kumar, Erin Schexnaydre, Karim Rafie, Tatsuaki Kurata, Ilya Terenin, Vasili Hauryliuk, Lars‐Anders Carlson

**Affiliations:** ^1^ Department of Medical Biochemistry and Biophysics Umeå University Sweden; ^2^ Wallenberg Centre for Molecular Medicine Umeå University Sweden; ^3^ Laboratory for Molecular Infection Medicine Sweden (MIMS) Umeå University Sweden; ^4^ Department of Experimental Medicine University of Lund Sweden; ^5^ Belozersky Institute of Physico‐Chemical Biology Lomonosov Moscow State University Russia; ^6^ Department of Molecular Biology Umeå University Sweden; ^7^ Institute of Technology University of Tartu Estonia

**Keywords:** COVID‐19, Nsp1, pathogenicity, ribosome, SARS‐CoV‐2, virus

## Abstract

Nonstructural protein 1 (Nsp1) of SARS‐CoV‐2 inhibits host cell translation through an interaction between its C‐terminal domain and the 40S ribosome. The N‐terminal domain (NTD) of Nsp1 is a target of recurring deletions, some of which are associated with altered COVID‐19 disease progression. Here, we characterize the efficiency of translational inhibition by clinically observed Nsp1 deletion variants. We show that a frequent deletion of residues 79–89 severely reduces the ability of Nsp1 to inhibit translation while not abrogating Nsp1 binding to the 40S. Notably, while the SARS‐CoV‐2 5′ untranslated region enhances translation of mRNA, it does not protect from Nsp1‐mediated inhibition. Finally, thermal stability measurements and structure predictions reveal a correlation between stability of the NTD and the efficiency of translation inhibition.

## Graphical abstract

SARS‐CoV‐2, the virus that causes COVID‐19, produces a protein called nonstructural protein 1 (Nsp1) whose task is to slow down protein production in the infected cell. In this paper, we show that several mutations in Nsp1 that have appeared in patients weaken its ability to shut down protein production, and we reveal the biochemical basis of this phenomenon.

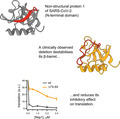

## Abbreviations

**COVID‐19**, coronavirus disease 2019

**CTD**, C‐terminal domain

**Nsp1**, nonstructural protein 1

**NTD**, N‐terminal domain

**pLDDT**, per‐residue prediction quality score

**SARS‐CoV‐2**, severe acute respiratory syndrome coronavirus 2

**TSA**, thermal shift assay

**UTR**, untranslated region

Coronaviruses (CoVs) are a family of enveloped, positive‐sense single‐stranded RNA viruses subdivided into four genera: Alpha‐, Beta‐, Gamma‐, and Deltacoronaviruses [1]. Four so‐called common‐cold coronaviruses that cause only mild disease are prevalently circulating in the human population: HCoV‐OC43, HCoV‐HKU1, HCoV‐229E, and HCoV‐NL63 [2]. The situation has changed dramatically in the recent years with the zoonotic introduction of three highly pathogenic Betacoronaviruses into humans: SARS‐CoV in 2002 [2], MERS‐CoV in 2012 [2], and, most recently, SARS‐CoV‐2 in 2019 [3, 4, 5, 6]. SARS‐CoV‐2 is the cause of the COVID‐19 pandemic which to date has caused nearly five million confirmed deaths (https://coronavirus.jhu.edu/map.html, retrieved 2021‐10‐21).

**Fig. 1:**
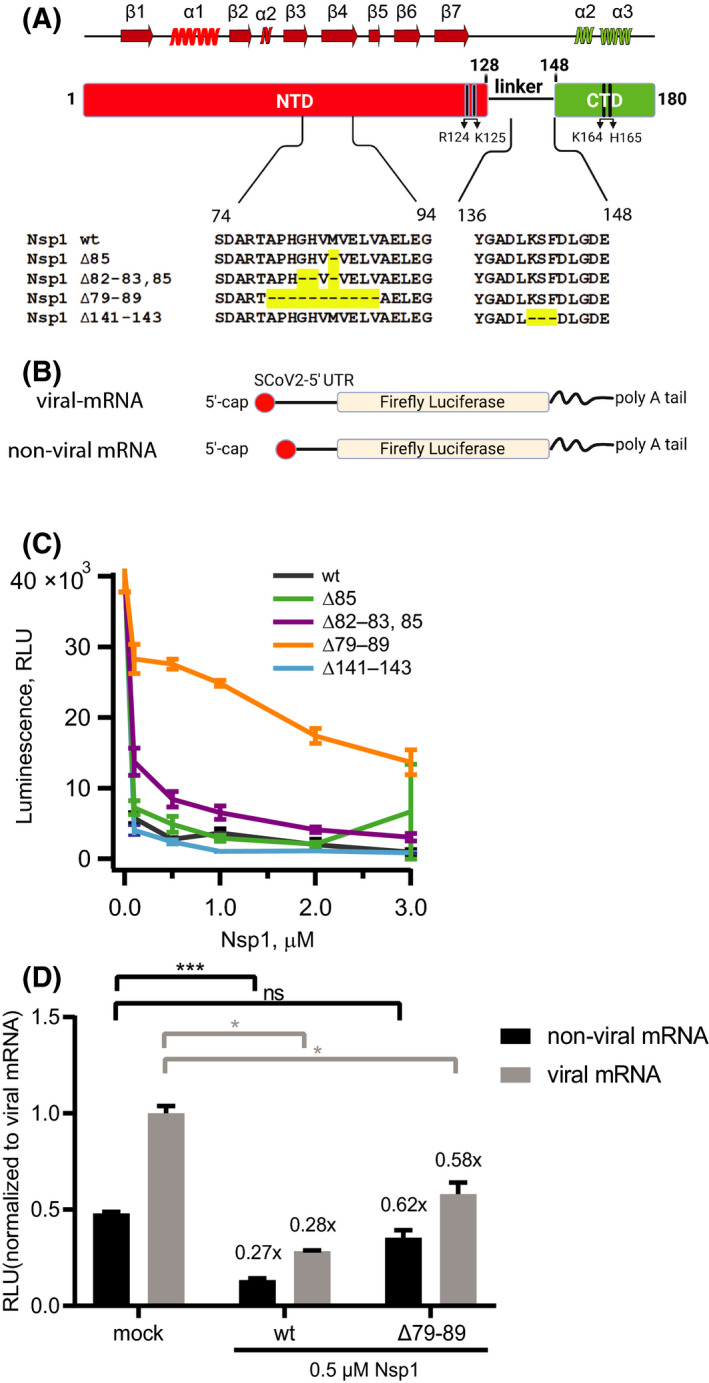
Nsp1 variants with amino acid deletions located outside of the mRNA tunnel‐targeting CTD differ in their ability to inhibit translation. (A) Structural organization of SARS‐CoV‐2 Nsp1 protein showing the N‐terminal and C‐terminal domains (NTD and CTD) as well as the regions containing naturally occurring deletions [25, 26]. Also denoted are amino acids R124‐K125 necessary for host mRNA cleavage [11] and the R164‐K165 pair reported to be necessary for the interaction with the 40S mRNA entry site [12]. The secondary structure elements of wild‐type Nsp1 are shown above the domain organization. (B) Schematic of the viral mRNA (mRNA reporter construct containing the 5′ UTR of SARS‐CoV‐2) and the nonviral mRNA (mRNA reporter construct with 5ʹ non‐SARS‐CoV‐2 UTR) followed by a firefly luciferase open reading frame, with 5′ cap and 3′ poly A tail. (C) Results of the *in vitro* translation assay to assess the translation efficiency of the reporter mRNA. Graph showing luminescence signal in response to firefly luciferase activity in HEK293F translational lysate at the end of the reaction, in the absence and presence of increasing concentrations (0.1, 0.5, 1.0, 2.0, and 3.0 μm) of recombinant Nsp1 and deletion variants (∆85; ∆82–83, 85; ∆79–89; ∆141–143). The average luminescence (RLU) from three independent experiments are shown in the plot, error bars represent standard deviation. (D) Comparison of luciferase activity measurements of wt Nsp1 and ∆79–89 against nonviral and viral reporter mRNAs. Relative luciferase activities were normalized to the activity of viral mRNA of mock sample (0 μm). Bars represents the mean value and error bars represent the std. *P* value of an unpaired *t*‐test (for viral mRNA—wt in comparison with mock *P* = 0.021, ∆79–89 in comparison with mock *P* = 0.023; for nonviral mRNA—wt in companion with mock *P* = 0.006, ∆79–89 in comparison with mock *P* = 0.124). *P < 0.05; ***P < 0.01.

Coronaviruses have unusually large genomes for positive‐sense single‐stranded RNA viruses. At 27–32 kb, they are an approximately threefold larger than other representatives of this group. This genomic expansion is thought to be made possible by a more complex, proofreading‐capable polymerase [7], and it has allowed coronaviruses to acquire a larger toolbox of host cell‐manipulating proteins. One such key pathogenicity factor is the nonstructural protein 1 (Nsp1). This protein has no known enzymatic activity, and while betacoronaviruses deleted for the *nsp1* gene can replicate in cell culture, they are strongly attenuated *in vivo* [8]. The 180 amino acid‐long SARS‐CoV Nsp1 (with which SARS‐CoV‐2 Nsp1 shares 84% sequence identity and displays conservation of all known key motifs) was found to suppress the antiviral host cell interferon response through a dual mechanism: it mediates the cleavage of host cell mRNAs by an unknown ribonuclease [9], and it suppresses translation through direct interaction with the small (40S) subunit of the ribosome [10]. Distinct mutations in SARS‐CoV *nsp1* have been described that selectively abrogate either of these two effects: substitutions of Arg124‐Lys125 in the folded N‐terminal domain of SARS‐CoV were shown to abolish the Nsp1‐induced mRNA cleavage [11], and residues Lys164‐His165 in the C‐terminal domain have been shown to be essential for binding to the 40S subunit of the ribosome (Fig. 1A) [12, 13]. SARS‐CoV‐2 Nsp1 binds empty small ribosomal subunit, as well as the 43S preinitiation complex and the full 80S ribosome, in all cases only efficiently engaging the open conformation of the small subunit [14]. In cryo‐EM structures of SARS‐CoV‐2 Nsp1 bound to the human 40S, the C‐terminal ~ 30 residues (148–180 in PDB ID 6ZLW) were present in a sufficiently rigid conformation that an atomic model could be built [15, 16, 17]. This model shows that the C‐terminal part of Nsp1 folds as two α‐helices that form a hairpin‐like arrangement inside the mRNA tunnel, incompatible with concomitant binding of mRNA. Consistent with the functionally crucial location of the C‐terminal domain in the Nsp1:40S complex, both its truncation (Δ118–180) and K164/H165A substitution abrogate the Nsp1 interaction with the small ribosomal subunit [18]. Off the ribosome the C‐terminal region (131–180) is unstructured, suggesting a possibility that the structure is attained upon recruitment to the ribosome [19]. The N‐terminal domain could be located in the cryo‐EM maps but at too low a resolution to allow model building, presumably due to flexibility with respect to the 40S. The cryo‐EM derived location of the Nsp1 N‐terminal domain in the ribosomal complex is further supported by *in situ* cross‐linking mass spectrometry identifying multiple crosslinks between Nsp1 and ribosomal protein S3 [18, 20].

The structure of Nsp1 bound to the ribosome raises the question how any translation is possible if Nsp1 blocks the mRNA channel. Somewhat paradoxically, experiments with *in vitro* translation systems have shown that SARS‐CoV‐2 Nsp1 can efficiently inhibit translation of both host and SARS‐CoV‐2 mRNAs, with some studies showing slightly smaller and others showing equal relative inhibition of viral mRNA [14, 17, 21]. However, with SARS‐CoV‐2 5′ untranslated region (UTR) acting as a translational enhancer, mRNAs with the viral 5′ UTR are inherently translated more efficiently than host mRNAs [17]. Therefore, even if translation of viral mRNA is inhibited to a similar relative degree by Nsp1, viral mRNA still has higher relative translational efficiency during infection. Furthermore, Tidu et al. [21] suggested that viral mRNA is less sensitive to inhibition by Nsp1, while similar degree of inhibition of viral and nonviral mRNA was reported by Schubert et al. [17] and Lapointe et al. [14]. An interaction between Nsp1 and the stem‐loop region 1 (SL1) of the viral 5′ UTR was implicated in partial escape of SARS‐CoV mRNAs from Nsp1 suppression [22], but the lack of affinity of SARS‐CoV‐2 Nsp1 for the viral 5′ UTR remains to be reconciled with such a mechanism [21]. The N‐terminal region of Nsp1 was shown to play a role in translational shutdown, and its deletion (Δ1–117) attenuating both ribosome and mRNA binding of SARS‐CoV‐2 Nsp1 [18].

In the course of the ongoing COVID‐19 pandemic, extensive genetic characterization has been carried out of new emerging mutants and virus variants. Nsp1 displays one of the highest degrees of diversity among SARS‐CoV‐2 proteins [23, 24, 25], with several circulating viruses with deletions in Nsp1 reported [25, 26]. A three‐residue deletion (Δ141–143) was described as appearing in clinical isolates from different geographical localities [26]. These three residues are located close to, albeit not in, the region which forms an ordered structure inside the 40S mRNA tunnel. It thus seems possible that this shortening of the linker region between the ribosome‐inserting part and the stably folded N‐terminal β‐barrel domain [27, 28] may affect the association of this altered protein with the ribosome, and thus its effectiveness in translational shutoff. Another cluster of deletions is located around residues 79–89 of Nsp1 [25]. This results in various length deletions around a loop in the atypical β‐barrel of the folded N‐terminal domain. The longest deletion, Δ79–89, is reported to be the fifth most common deletion in SARS‐CoV‐2 Nsp1 in a worldwide comparison [25]. The 79–89 deletion, as well as shorter deletions in the same region of Nsp1, correlates with higher cycle threshold (Ct) values in patients (i.e. lower viral load), less severe disease outcome, and a weaker interferon response as measured by lower serum levels of IFN‐β [25]. The biochemical basis for the altered disease course associated with Nsp1 deletions is not established. Here, we purified a number of Nsp1 proteins corresponding to these circulating mutations in SARS‐CoV‐2 and compared their potency in inhibiting translations using a human cell *in vitro* translation lysate. We correlate the findings to thermal stability assays and protein fold predictions of the mutants. The result shed light on the clinical and cellular findings related to Nsp1‐mutated SARS‐CoV‐2 isolates.

## Materials and methods

### Cloning and mutagenesis

For expression of wild‐type Nsp1, the *nsp1* gene was amplified from the construct pLVX‐EF1alpha‐SARS‐CoV‐2‐nsp1‐2xStrep‐IRES‐Puro (Addgene) [29] and inserted into a 1B vector (Macrolab, UC Berkeley) using In‐Fusion cloning kit (Takara Biosciences, San Jose, CA, USA). Deletion mutations were generated from this plasmid by standard site‐directed mutagenesis methods. The reporter plasmid T7‐5′SARS‐CoV‐2 UTR‐Firefly luciferase was generated by inserting the T7 promoter upstream of SARS‐CoV2 5′ UTR fused to the firefly luciferase coding sequence (amplified from T7‐cmvtrans‐Ffluc‐poly A) into the destination vector pUC19. The reporter plasmid with nonviral UTR was also based on the plasmid T7‐cmvtrans‐Ffluc‐poly A (Addgene). All plasmids were sequenced to confirm cloning of the correct sequence.

### Protein expression and purification

Wild‐type Nsp1 and all deletion variants were expressed and purified as follows. The plasmid was transformed into *Escherichia coli* BL21 (DE3) cells for overexpression. An overnight culture was grown at 37 °C to inoculate the secondary culture. Cells were grown at 37 °C until the OD_600_ reached 0.4, after which the incubator temperature was changed to 25 °C to let the cells cool down to induction temperature 25 °C. At OD_600_ of around 0.8–0.9 the protein expression was induced by addition of 0.5 mm Isopropyl β‐d‐1‐thiogalactopyranoside (IPTG) and the protein was expressed at 25 °C overnight. Cells were harvested by centrifugation at 7000 ***g*** (rotor JLA‐8.1000; Beckman Coulter, Brea, CA, USA) for 60 min. After discarding the supernatant, the cell pellet was washed with lysis buffer (50 mm HEPES‐NaOH, pH 7.4, 300 mm NaCl, 0.1 mm THP, 10 mm imidazole, and 5% glycerol) and stored at −80 °C.

Cell mass was thawed and resuspended in lysis buffer supplemented with DNase I and protease inhibitor cocktail (1 mm benzamidine, 0.2 mm phenylmethylsulfonyl fluoride, and 5 µm leupeptin). Homogenized suspension was then passed twice through a cell disruptor (Constant System Limited, Daventry, UK) at a pressure 27 kPsi. The lysate was clarified by centrifugation at 36 000 ***g*** (rotor JA‐25.50; Beckman Coulter) for 1 h, and the supernatant passed through a 0.22‐μm syringe filter. One millilitre Ni‐Sepharose Fastflow resin (Cytiva, Umeå, Sweden) pre‐equilibrated with lysis buffer was combined with clarified lysate and incubated for 2 h at 4 °C under gentle agitation. Next, the resin was loaded onto a gravity‐flow column. The protein‐bound resin was washed twice with 20 mL wash buffer (50 mm HEPES‐NaOH, pH 7.4, 300 mm NaCl, 0.1 mm THP, 30 mm Imidazole, and 5% glycerol) twice. The resin was then resuspended in 4 mL lysis buffer, supplemented with TEV protease (approx. 70 μg·mL^−1^) and incubated overnight at 4 °C on a rotator wheel. The cleaved protein was collected as flowthrough. An additional wash with 5 mL lysis buffer was performed to collect the residual cleaved protein. For production of His_6_‐tagged Nsp1, the protein was instead eluted from the resin using wash buffer supplemented with 250 mm imidazol. The proteins were then further purified by anion exchange chromatography. To do so, after diluting with buffer A (50 mm HEPES‐NaOH, pH 7.4, 100 mm NaCl, 0.1 mm THP, and 5% glycerol), diluted sample was filtered using 0.22‐μm syringe filter (VWR, Radnor, PA, USA) and loaded onto a HiTrap Q HP 1ml column (Cytiva, Umeå, Sweden). The sample was eluted with a gradient going from buffer A to buffer B (50 mm HEPES‐NaOH, pH 7.4, 1 m NaCl, 0.1 mm THP, and 5% glycerol) in 14 mL. Nsp1‐containing fractions were pooled and concentrated using a Vivaspin 6 centrifugal unit with 5 kDa cut off membrane (EMD Millipore, Burlington, MA, USA) before being loaded onto a Superdex 75 increase 10/300 size‐exclusion column (Cytiva) that was pre‐equilibrated with SEC buffer (20 mm HEPES‐KOH, pH 7.51, 200 mm KOAc, 2 mm Mg(OAc)_2_, 0.1 mm THP, and 2.5% glycerol). Protein elutions were subjected to SDS/PAGE gel electrophoresis and the fractions corresponding to the center of the Nsp1‐containing peak were pooled and concentrated. Aliquots were then flash frozen in liquid N_2_ and stored at −80 °C. The identity of all the proteins was confirmed by trypsin‐digestion mass spectrometry. His_6_‐tagged protein was used for western blotting of density gradient fractions, whereas all other experiments were done with untagged protein.

### *In vitro* transcription

Capped and polyadenylated RNA transcripts were synthesized from linearized plasmid (T7–5ʹ‐SCoV2‐UTR–firefly Luciferase) using the mMESSAGE mMACHINE T7 Ultra kit (ThermoFisher, Watham, MA, USA) following the manufacturer protocol. Briefly, all the reagents including the 5ʹcap analog were gently mixed with linearized plasmid and transcription was performed by T7 RNA polymerase at 37 °C for 2 h. Then, 1 μL of Turbo DNase was gently mixed with the transcription mixture and incubated further at 37 °C for 15 min. In order to add poly (A) tail to the 3ʹ end of the *in vitro* transcribed RNA, *E. coli* Poly(A) Polymerase I (E‐PAP) was added along with other provided reagents to the transcription mixture and after mixing gently incubated at 37 °C for 45 min. According to the manufacturer, this poly(A) tailing adds at least 150 adenines to the 3ʹ end of the mRNA. Finally, the RNA prep was recovered by lithium chloride precipitation and quality‐checked by running a sample on a denaturing agarose gel, which resulted in a single band. The capped and poly(A)‐tailed RNA was aliquoted in 10 μL volumes and flash frozen in liquid N_2_ and stored at −80 °C. For simplicity, the reporter mRNA with SARS‐CoV‐2 5′ UTR is referred to as ‘viral mRNA’ and the one with non‐SARS‐CoV2 5′ UTR is referred to as ‘nonviral mRNA’ throughout the text.

### Preparation of HEK293F translation lysate

*In vitro* translation lysates were prepared from HEK293F cells using a previously described protocol [30, 31, 32]. Cells were scraped and collected by centrifugation for 5 min at 600 r.p.m. at 4 °C. Cells were washed once with cold PBS (137 mm NaCl, 2.7mm KCl, 100mm Na_2_HPO_4_, 2mm KH_2_PO_4_) and resuspended in Lysolecithin lysis buffer (20 mm HEPES‐KOH, pH 7.4, 100 mm KOAc, 2.2 mm Mg(OAc)_2_, 2 mm DTT, and 0.1 mg·mL^−1^ lysolecithin), using 1 mL for 8 × 10^6^ cells. Cells were incubated for 1 min on ice, then immediately centrifuged for 10 s at 10 000 ***g*** at 4 °C. The pellet was resuspended in cold hypotonic extraction buffer (20 mm HEPES‐KOH, pH 7.5, 10 mm KOAc, 1 mm Mg(OAc)_2_, 4 mm DTT, and Complete EDTA‐free protease inhibitor cocktail (Roche, Basel, Switzerland) at an equal volume to the size of the pelleted cells. After 5 min of incubation on ice, cells were transferred into a precooled Dounce homogenizer and lysed by 20–25 strokes. The lysate was centrifuged at 10 000 ***g*** for 10 min at 4 °C, and the supernatant transferred to a fresh tube. Aliquots were flash frozen in liquid N_2_ and stored at −80 °C.

### *In* *vitro* translation assays

*In vitro* translation reactions were performed as previously described [30, 31, 32], with modifications relating to the Nsp1 addition. HEK293F‐cell lysate was pre‐incubated with increasing concentrations (from 0 to 3 μm final concentration) of recombinant Nsp1 (wild‐type and variants) for 15 min on ice. Translation buffer (20 mm HEPES‐KOH, pH 7.6, 1 mm DTT, 0.5 mm spermidine‐HCl, 1 mm Mg(OAc)_2_, 8 mm creatine phosphate, 1 mm ATP, 0.2 mm GTP, 150 mm KOAc, 25 μm of each amino acid, and 2 units of human placental ribonuclease inhibitor (Fermentas, Burlington, Canada) was then added followed by 1 μL of the reporter mRNA (0.5 pmol·μL^−1^) to give a total reaction volume of 13 μL. Final RNA concentration in the reaction mixture was kept at 38 nm. Translation reactions were incubated at 30 °C for 3 h, samples were flash frozen in liquid N_2_ and kept at −80 °C. Luciferase assays were performed using the Steady‐Glo Luciferase Assay kit (Promega, Madison, WI, USA) following the manufacturer's protocol. Luminescence was measured using the M200 finite series microplate reader (TECAN, Männedorf, Switzerland). Samples for all concentrations of each protein were prepared in triplicates and measured.

### Sucrose gradient fractionation and western blotting

20 A_260_ units of HEK293F translation lysate were supplemented with either wild‐type or Δ79–89 variant N‐terminally His‐tagged Nsp1 (final concentration of 1 µm) in hypotonic extraction buffer or just hypotonic extraction buffer and kept on ice for 15 min. One hundred microlitre or the resultant mixture was loaded onto 10–50% sucrose gradient in HEPES:Polymix buffer pH 7.5 (5 mm Mg^2+^ final concentration) [33] and resolved by centrifugation at 202 000 ***g*** for 2.5 h at 4 °C without braking in a SW‐41Ti rotor (Beckman Coulter rotor). Gradients were fractionated using Biocomp Gradient Station (BioComp Instruments, Fredericton, Canada) with A_260_ as a readout.

For rRNA electrophoresis 8 µL fractions were supplemented with 2 mL of 5× loading buffer (8.3 mm Tris/HCl pH 7.6, 1% SDS (w/v), 0.025% Bromophenol blue, 0.025% xylene cyanol FF, 0.125% orange G, 50% glycerol (w/v) 60 mm EDTA), resolved on 1.5% agarose gel and stained with SYBR Gold (Life Technologies, Carlsbad, CA, USA; S11494) and imaged using Amersham™ ImageQuant™ 800 imaging system (Cytiva).

For western blotting 0.5 mL fractions were supplemented with 1.5 mL of 99.5% ethanol and precipitated overnight at −20 °C. After centrifugation at 21 000 ***g*** for 30 min at 4 °C the supernatants were discarded and the samples were dried. The pellets were resuspended in 30 µL of 2× SDS loading buffer (100 mm Tris/HCl pH 6.8, 4% SDS (w/v) 0.02% Bromophenol blue, 20% glycerol (w/v) 4% β‐mercaptoethanol), resolved on the 12% SDS/PAGE and transferred to nitrocellulose membrane (Trans‐Blot Turbo Midi Nitrocellulose Transfer Pack, Bio‐Rad, 0.2 µm pore size) with the use of a Trans‐Blot Turbo Transfer Starter System (Bio‐Rad, Hercules, CA, USA) (5 min at 1.3 A, 25 V). Membrane blocking was done for 1 h in PBS‐T (1× PBS 0.05% Tween‐20) with 5% w/v nonfat dry milk at room temperature. Nsp1 was detected using anti‐penta‐His antibody (QIAGEN, Hilden, Germany, 34660; 1 : 5000 dilution) primary combined with Goat anti‐Mouse IgG‐HRP (Agrisera, Vännäs, Sweden, AS111772; 1 : 5000 dilution) as well as Can Get Signal™ immunostain (TOYOBO, Osaka, Japan). ECL detection was performed using WesternBright™ Quantum (K‐12042‐D10; Advansta, San Jose, CA, USA) Western blotting substrate and aAmersham™ ImageQuant™ 800 imaging system (Cytiva).

### Thermal shift assay

All proteins were used at a final concentration of 1 mg·mL^−1^ in a total volume of 20 μL. SYPRO Orange (Invitrogen S6651) was used at a final concentration of 5× for all the samples. Experiments were carried out in 20 mm HEPES‐KOH, pH 7.51, 200 mm KOAc, 2 mm Mg (OAc)_2_, 0.1 mm THP, and 2.5% glycerol. Each sample was prepared in triplicates. Samples were dispensed into FrameStar 96‐well PCR plate (4titudE 4ti‐07 10/C) sealed afterward with PCR optical Seal (4titudE). Thermal scanning (10–95 °C at 1.5 °C·min^−1^) was performed using a real‐time PCR instrument C1000 Touch Thermal Cycler (CFX96 from Bio‐Rad) and fluorescence intensity was measured after every 10 s. According to the described protocol, raw data were truncated in Microsoft Excel to remove postpeak quenching [34]. A nonlinear fitting of the truncated dataset to a Boltzmann Sigmoidal equation was performed to obtain the melting temperature (*T*_m_) using prism 9 (GraphPad Software, San Diego, CA, USA).

### Structure predictions

All structure predictions were performed using alphafold2 [35] as implemented in colabfold (https://colab.research.google.com/github/sokrypton/ColabFold/blob/main/AlphaFold2.ipynb) with default settings and allowing the structures to relax using the built‐in amber [36] functionality.

### Molecular graphics and visualization

Cartoon representations of Nsp1 structures were generated using chimerax [37]. Schematic representations of structure topology were drawn in topdraw [38].

## Results

### Circulating deletions in SARS‐CoV‐2 Nsp1 differ in their inhibitory effect on translation

Several amino acid deletions are reported in SARS‐CoV‐2 Nsp1: Δ85, Δ82–83, 85 and Δ79–89 in the N‐terminal domain (NTD) [25] and Δ141–143 in the C‐terminal domain (CTD) [26] (Fig. 1A). These deletions have been correlated to clinical characteristics, but the mechanistic biochemical basis of why they alter the course of COVID‐19 has not been determined. We wished to assess the effect of these deletions on Nsp1's ability to shut down host translation. As a first step toward this analysis, we purified wild‐type Nsp1 and a series of deletion mutants to homogeneity and monodispersity (Fig. S1). We then established a translation assay based on lysates of the human cell line HEK293F. Since Nsp1 has been reported to reduce translation of viral mRNAs to a lesser degree than cellular mRNAs [14, 17, 21], we reasoned that a reporter mRNA resembling a viral mRNA would provide a more sensitive assay. Thus, we measured translation efficiency of a reporter mRNA encoding firefly luciferase, equipped with the SARS‐CoV‐2 5′ UTR, a 5′ cap, and 3′ poly(A) sequence, in the presence of increasing concentrations of both wild‐type recombinant Nsp1 and its deletion variants (Fig. 1B).

**Fig. 2:**
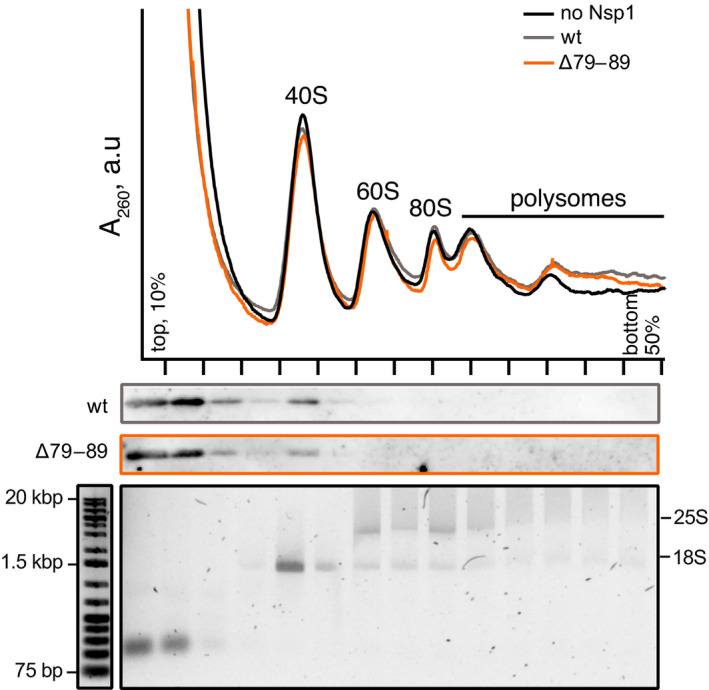
Deletion of residues 79–89 does not abrogate Nsp1 binding to 40S ribosomal subunit. HEK293F translation lysate was combined with His_6_‐tagged Nsp1 protein (wt or Δ79–89) and resolved by ultracentrifugation on a 10–50% sucrose gradient. rRNA and His_6_‐Nsp1 protein in individual fractions were visualized using agarose electrophoresis and western blotting, respectively.

In agreement with earlier reports [17, 21], wild‐type Nsp1 efficiently abrogates production of firefly luciferase in concentration‐dependent manner. Already at 0.1 μm, wild‐type (wt) protein reduces the translational efficiency by more than 95% (Fig. 1C). An effect similar to the wt is observed for the NTD deletion variant ∆85 and the CTD deletion variant ∆141–143. The NTD deletion variant ∆82–83, 85 shows slightly reduced suppression of translation compared to the wild‐type. In contrast, the longest deletion in the NTD, ∆79–89, is substantially weakened in its ability to inhibit translation as compared to wt Nsp1 (Fig. 1C). We observed that even at 3 μm protein concentration, the translation efficiency reduces only to 40–50%, establishing that the ∆79–89 protein is less effective in translation shutdown than the wild‐type and other deletion variants. Notably, the ∆79–89 protein variant is still able to bind the human 40S ribosomal subunit (Fig. 2).

Having investigated how Nsp1 variants inhibit the translation of viral mRNA, we next turned to the question of whether they are differentially efficient in inhibiting viral and nonviral (host) mRNA. We thus repeated the translation assay with mRNAs having either SARS‐CoV‐2 or a nonviral 5′ UTR (Fig. 1B). Consistent with previous report by Schubert et al. [17], the viral mRNA was more strongly translated in the absence of Nsp1 (Fig. 1D). We then repeated this assay in the presence of wt and ∆79–89 Nsp1, both used at 0.5 μm final concentration. Both Nsp1 variants had the same relative effect on translation of viral and nonviral mRNA, thus leading to a higher absolute level of translation of the viral mRNA in presence of either Nsp1 (Fig. 1D). Note that the experiments presented in Fig. 1C,D were conducted with different batches of translation lysate. They can thus be qualitatively, but not quantitatively compared to each other. Taken together, these data establish a biochemical basis for correlating COVID‐19 disease progression to the translational shutdown efficiency of circulating deletion mutants in Nsp1.

### Deletions in Nsp1 lead to altered protein stability that correlates with translation inhibition

**Fig. 3:**
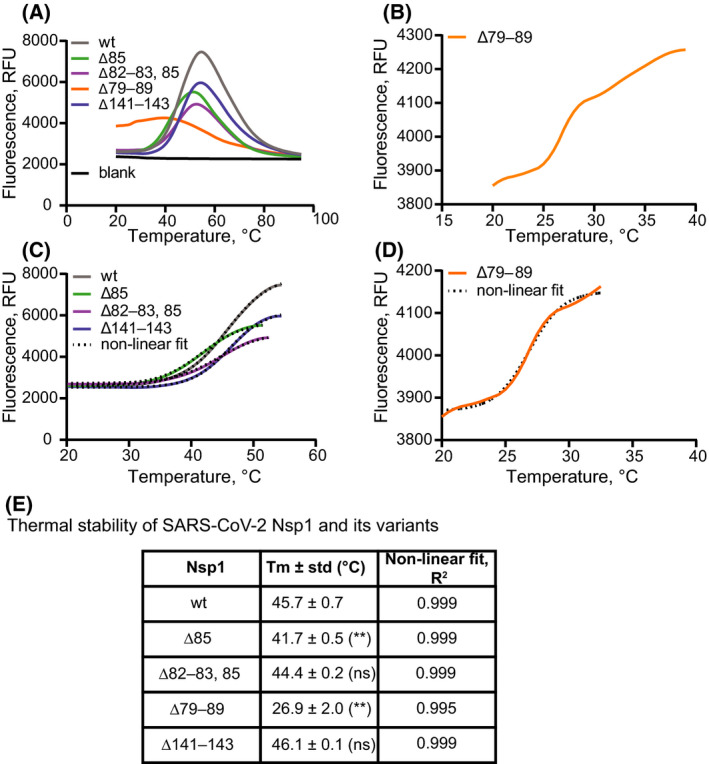
The ∆79–89 deletion variant of SARS‐CoV2 Nsp1 has a drastically reduced thermal stability. (A) Raw data of thermal shift assays [34] with Nsp1 variants using SYPRO Orange. (B) Raw TSA data of the deletion variant ∆79–89. (C) Nonlinear regression curve fit (Boltzmann sigmoidal) of the unfolding curves of Nsp1 variants (except that of the ∆79–89 protein) to calculate the melting temperature. (D) Same as (C), for the ∆79–89 variant. (E) Tabular presentation of the result of the nonlinear fitting of the unfolding curve of all the proteins. Statistical significance of *T*_m_ change from wt was estimated for each deletion variants by unpaired t test and is indicated in the table. *P* value for ∆85 = 0.002, for ∆82–83, 85 = 0.066, for ∆79–89 = 0.002, and for ∆141–143 = 0.423. n.s., not significant. **P < 0.01.

None of the investigated *nsp1* mutations was in the region previously reported to interact with the ribosome, and yet the longer deletions in the NTD were clearly altered in their translation shutdown capacity. We wanted to investigate whether this effect could be due to the destabilizing effects of the deletions on the NTD. We first noted that all proteins were soluble and monodisperse when purified from *E. coli*, but the Δ79–89 construct eluted at a lower volume in size exclusion chromatography, indicting a larger hydrodynamic radius (Fig. S1). To probe the effects of the deletions on protein stability, we assessed the thermal stability of wt Nsp1 and deletion variants by thermal shift assay (TSA) [39] (Fig. 3). In this assay, SYPRO orange dye is used as a probe to estimate the extent of unfolding of the protein with increasing temperature, and the melting temperature (*T*_m_) from each curve is determined as described in the experimental procedure section. Raw TSA data (Fig. 3A,B) demonstrate that all mutants of SARS‐CoV‐2 Nsp1 showed similar, wild‐type‐like unfolding profile, apart from the longest deletion variant (∆79–89). Wt Nsp1 and all altered proteins, with a notable exception of ∆79–89, allowed for fitting of a simple melting curve (Fig. 3C) indicating a single transition temperature that is comparable to that of the wt protein (Fig. 3E). The thermal denaturation curve of ∆79–89 (Fig. 3A,B) was shallow and could not be perfectly recapitulated by fit to a single melting temperature (Fig. 3D). However, using the first part of the curve gave a fit that is consistent with the qualitative appearance of the curve (Fig. 3D) and a lowered thermal stability. This suggests that already at room temperature the structural integrity of ∆79–89 variant is compromised. In summary, our results establish a correlation between structural stability of Nsp1's NTD and its ability to inhibit translation.

### Structure predictions suggest that NTD β‐barrel destabilization causes the decreased stability of Δ79–89 nsp1

**Fig. 4:**
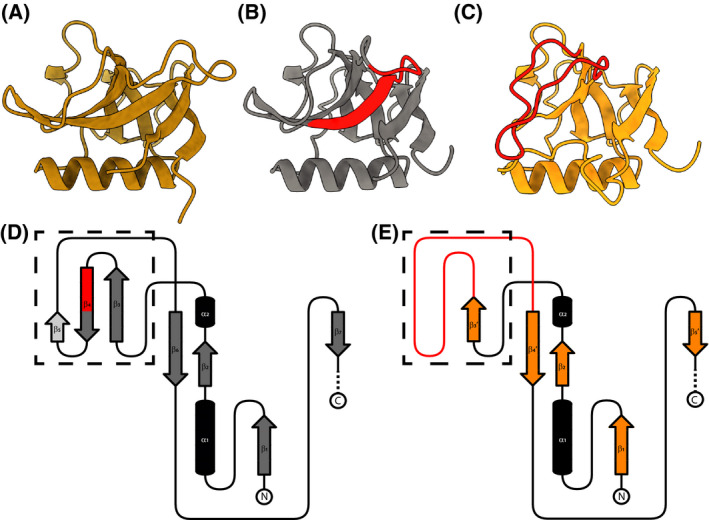
Predicted β‐barrel integrity correlates with altered thermal stability of Nsp1 deletion variants. (A) Cartoon representation of the experimentally determined structure of the Nsp1 N‐terminal β‐barrel domain (PDB ID: 7K7P, [27]). (B) Cartoon representation of the predicted wt Nsp1 structure (gray), truncated to the same boundaries as for (A). In red highlighted are residues 79–89. (C) Cartoon representation of the predicted Δ79–89 Nsp1 structure (orange), truncated to the same boundaries as for (A). In red highlighted is the disordered region, which in (B) forms the β‐strands β3–β5. (D) Topological drawing of the wild‐type Nsp1 fold with α‐helices shown as black tubes and the β‐strands as arrows. β‐strands forming the β‐barrel are shown in gray and non‐β‐barrel forming strands in light gray. Highlighted in red is the β‐strand part deleted in Δ79–89 Nsp1. (E) Topological drawing of the Δ79–89 Nsp1 fold. α‐helices are shown as black tubes and the β‐strands as orange arrows. Highlighted in red is the disordered region as in (C). (D, E) Dashed‐lined box highlights the location of change between the predicted wt and Δ79–89 folds.

We next wanted to explore the possible structural basis for the decreased translation inhibition and thermal stability of the altered Nsp1 proteins. To investigate how the Nsp1 deletions impact its structure we predicted the structures of wt, Δ141–143, Δ85, Δ82–83, 85, and Δ79–89 Nsp1 using alphafold 2 [35]. Since there are available experimental structure of the free SARS‐CoV‐2 Nsp1 NTD (residues 10–126) [27], as well as the ribosome‐inserted CTD [15, 16, 17], we could compare them to the prediction of the wt Nsp1 structure as a baseline. The predicted Nsp1 structure aligns very well with the experimentally determined N‐terminal β‐barrel domain, with average positional shifts (root‐mean‐square deviation, RMSD) of the respective Cα atoms of only 0.64 Å for residues 10–126 (Fig. 4A, Fig. S2A). In the CTD, two α‐helices are correctly predicted where they are observed in the experimental structure, albeit at a different angle to each other than in the 40S‐bound structures (Fig. S2A). Overall, the per‐residue prediction quality score (pLDDT) correlated with the rigidity of the fold across Nsp1, with the NTD β‐barrel having higher scores than the CTD which was reported to be flexible in solution (Fig. S2B) [19]. Supported by the accurate prediction of the wt Nsp1 structure, we thus reasoned that structure predictions for the altered Nsp1 proteins may shed light on the differential effects of the deletions on protein stability. Unsurprisingly, the deletion Δ141–143, predicted to be located in a disordered region of the CTD, had no effect on the predicted fold of other parts of Nsp1 (Fig. S2). The other three deletions (Δ85, Δ82–83, 85, and Δ79–89) are all located at the beginning of the fourth β‐strand of the β‐barrel (β4) (Fig. 4B,D). Interestingly, despite being located in a secondary structure element, the two shorter deletions are not predicted to affect the β‐barrel integrity (Fig. S2C), likely given that the hydrogen bonding between β‐strands is mediated by the protein backbone and can be rescued by the residues ‘next‐in‐line’ to the deleted residues. The predicted integrity of the fold of these deletions correlates well with their unaltered thermal stability (Fig. 3E). The only striking effect on the integrity of the β‐barrel domain is predicted to stem from the longest deletion. The Δ79–89 structure prediction suggests a near‐complete dissolution of the β3–β5 strands leading to a break in the β‐barrel domain, severely affecting its integrity (Fig. 4C,E). This prediction is in line with the drastic reduction in thermal stability for the Δ79–89 protein from 46 °C to ~ 27 °C (Fig. 3E). Taken together, structure predictions of the Nsp1 deletions suggest destabilization of the N‐terminal β‐barrel domain as the mechanism behind the altered properties of the Δ79–89 Nsp1 protein.

## Discussion

The high transmissibility of SARS‐CoV‐2 and the lack of pre‐existing immunity contributed to an explosive development of the COVID‐19 pandemic. This happening in the age of readily available RNA sequencing has given a fine‐grained view of the mutations taking place in the viral genome in the course of the pandemic. The nonstructural protein Nsp1 is a major pathogenicity factor for coronaviruses [8]. Although Nsp1 has remained unaltered in the major SARS‐CoV‐2 variants, certain internal deletions in Nsp1 (Fig. 1A) are regularly detected in clinical isolates and have been correlated to different clinical outcomes [25, 26]. The repeated appearance of certain deletions indicates that they are either better tolerated without losing infectivity, or that they are more likely to appear due to peculiarities of the RNA replication process. In this study, we used a human translation lysate assay to compare the potency of these Nsp1 deletion mutants in inhibiting translation. Notably, none of the deletions encompassed the two motifs previously identified as being necessary for Nsp1's ability to inhibit translation [12], and mediate mRNA degradation [11], respectively (Fig. 1A). Thus, it was not clear *a priori* what their effect on translation would be, if any. The main finding of this study is that several of these circulating Nsp1 deletion mutants are still highly potent in inhibiting translation *in vitro*, except for the longest deletion, Δ79–89. Whereas still soluble and monodisperse, the Δ79–89 protein was no longer able to shut down translation completely, even when present at thirtyfold higher concentrations than the wild‐type protein. We show that Δ79–89 Nsp1 also stands out from the other deletion mutants by having severely reduced thermal stability. Structure predictions of all mutants were consistent with this and suggested that the mechanism for the lost stability of the Δ79–89 protein is a rearrangement leading to a disruption of its N‐terminal β‐barrel domain fold.

The deletions around residues 79–89 in Nsp1 were previously characterized in terms of their effect on transcriptome and type I interferon response [25]. A side‐by‐side comparison of those results with our translation shutdown data reveals some interesting differences. Strikingly, the shorter deletions in this region already alter the transcriptome and lead to a much weakened type I IFN response as compared to the 180 residue version of Nsp1. These shorter deletions are not impaired in translation shutdown at the concentrations tested, indicating that this region of Nsp1 may have other functions. Notably, the deletions flank the only long loop connecting two strands of the β‐barrel. This loop was reported to be flexible in the NMR structure of SARS‐CoV Nsp1 [28], but it is to date unknown whether is involved in interactions with host or viral components.

In summary, we determined the translation shutdown capacity of several clinically detected deletions in SARS‐CoV‐2 Nsp1. The data show that a compromised stability of the N‐terminal β‐barrel abrogates translational shutdown, and further indicates that the shorter deletions around residue 79–89 may affect interferon response and transcriptome through a mechanism independent of translation shutdown.

## Author contributions

IT, VH, LAC conceived the study; PK, ES, KR, TK performed experiments; PK, KR, TK, IT, VH, LAC analysed data; PK, KR, VH, LAC wrote the initial manuscript; all authors read and edited the manuscript.

## Supporting information


**Fig. S1**. Purification of wild type and deletion variants of SARS‐CoV‐2 Nsp1. (A‐E) Chromatograms (280 nm absorption) of Nsp1 and its deletion variants eluting from a Superdex 75 increase 10/300 size‐exclusion column. Each chromatogram has the volume corresponding to the center of the main peak noted. The A260/A280 ratio was similar (~0.6) in the main peak of all constructs. (F) 1.5 μg of each protein sample was analyzed on a 15% SDS‐PAGE and stained with Coomassie blue.

**Fig. S2**. AlphaFold2‐predicted Nsp1 structures. (A) (left) Structures of the experimentally determined N‐ (residues 10‐126, PDBID: 7K7P[27]) and C‐terminal (residues 148‐180 PDBID: 6ZOJ[17]) domains of Nsp1. Structures are oriented as the predicted full‐length structure of wt Nsp1 in (B). (right) Superposition of the predicted wt Nsp1 structure with the experimentally determined structures as in (A). (B) Predicted structure of full length wt Nsp1 shown in cartoon representation (grey) and the corresponding per‐residue confidence score (pLDDT) trace for all five prediction runs. (C) Cartoon representations of the predicted structures of Δ141‐143 (light blue), Δ85 (green), Δ82‐83,85 (purple) and Δ79‐89 (orange) Nsp1 and their respective per‐residue confidence score (pLDDT) trace for each of their predictions.

## Data Availability

The data that support the findings of this study are available from the corresponding author (lars-anders.carlson@umu.se) upon reasonable request.

## References

[1] Drexler JF , Corman VM , Drosten C . Ecology, evolution and classification of bat coronaviruses in the aftermath of SARS. Antiviral Res. 2014;101:45–56. 24184128 10.1016/j.antiviral.2013.10.013 PMC7113851

[2] Corman VM , Muth D , Niemeyer D , Drosten C . Hosts and sources of endemic human coronaviruses. Adv Virus Res. 2018;100:163–88. 29551135 10.1016/bs.aivir.2018.01.001 PMC7112090

[3] Wu F , Zhao S , Yu B , Chen YM , Wang W , Song ZG , et al. A new coronavirus associated with human respiratory disease in China. Nature. 2020;579:265–9. 32015508 10.1038/s41586-020-2008-3 PMC7094943

[4] Chan JF , Yuan S , Kok KH , To KK , Chu H , Yang J , et al. A familial cluster of pneumonia associated with the 2019 novel coronavirus indicating person‐to‐person transmission: a study of a family cluster. Lancet. 2020;395:514–23. 31986261 10.1016/S0140-6736(20)30154-9 PMC7159286

[5] Huang C , Wang Y , Li X , Ren L , Zhao J , Hu Y , et al. Clinical features of patients infected with 2019 novel coronavirus in Wuhan, China. Lancet. 2020;395:497–506. 31986264 10.1016/S0140-6736(20)30183-5 PMC7159299

[6] Lu R , Zhao X , Li J , Niu P , Yang B , Wu H , et al. Genomic characterisation and epidemiology of 2019 novel coronavirus: implications for virus origins and receptor binding. Lancet. 2020;395:565–74. 32007145 10.1016/S0140-6736(20)30251-8 PMC7159086

[7] Robson F , Khan KS , Le TK , Paris C , Demirbag S , Barfuss P , et al. Coronavirus RNA proofreading: molecular basis and therapeutic targeting. Mol Cell. 2020;80:1136–8. 33338403 10.1016/j.molcel.2020.11.048 PMC7833706

[8] Zust R , Cervantes‐Barragan L , Kuri T , Blakqori G , Weber F , Ludewig B , et al. Coronavirus non‐structural protein 1 is a major pathogenicity factor: implications for the rational design of coronavirus vaccines. PLoS Pathog. 2007;3:e109. 17696607 10.1371/journal.ppat.0030109 PMC1941747

[9] Kamitani W , Narayanan K , Huang C , Lokugamage K , Ikegami T , Ito N , et al. Severe acute respiratory syndrome coronavirus nsp1 protein suppresses host gene expression by promoting host mRNA degradation. Proc Natl Acad Sci USA. 2006;103:12885–90. 16912115 10.1073/pnas.0603144103 PMC1568942

[10] Kamitani W , Huang C , Narayanan K , Lokugamage KG , Makino S . A two‐pronged strategy to suppress host protein synthesis by SARS coronavirus Nsp1 protein. Nat Struct Mol Biol. 2009;16:1134–40. 19838190 10.1038/nsmb.1680 PMC2784181

[11] Lokugamage KG , Narayanan K , Huang C , Makino S . Severe acute respiratory syndrome coronavirus protein nsp1 is a novel eukaryotic translation inhibitor that represses multiple steps of translation initiation. J Virol. 2012;86:13598–608. 23035226 10.1128/JVI.01958-12 PMC3503042

[12] Narayanan K , Huang C , Lokugamage K , Kamitani W , Ikegami T , Tseng CT , et al. Severe acute respiratory syndrome coronavirus nsp1 suppresses host gene expression, including that of type I interferon, in infected cells. J Virol. 2008;82:4471–9. 18305050 10.1128/JVI.02472-07 PMC2293030

[13] Shen Z , Zhang G , Yang Y , Li M , Yang S , Peng G . Lysine 164 is critical for SARS‐CoV‐2 Nsp1 inhibition of host gene expression. J Gen Virol. 2021;102:jgv001513. 10.1099/jgv.0.001513 PMC8116783 33151142

[14] Lapointe CP , Grosely R , Johnson AG , Wang J , Fernandez IS , Puglisi JD . Dynamic competition between SARS‐CoV‐2 NSP1 and mRNA on the human ribosome inhibits translation initiation. Proc Natl Acad Sci USA. 2021;118:e2017715118. 33479166 10.1073/pnas.2017715118 PMC8017934

[15] Yuan S , Peng L , Park JJ , Hu Y , Devarkar SC , Dong MB , et al. Nonstructural protein 1 of SARS‐CoV‐2 is a potent pathogenicity factor redirecting host protein synthesis machinery toward viral RNA. Mol Cell. 2020;80:1055–66.e6. 33188728 10.1016/j.molcel.2020.10.034 PMC7833686

[16] Thoms M , Buschauer R , Ameismeier M , Koepke L , Denk T , Hirschenberger M , et al. Structural basis for translational shutdown and immune evasion by the Nsp1 protein of SARS‐CoV‐2. Science. 2020;369:1249–55. 32680882 10.1126/science.abc8665 PMC7402621

[17] Schubert K , Karousis ED , Jomaa A , Scaiola A , Echeverria B , Gurzeler LA , et al. SARS‐CoV‐2 Nsp1 binds the ribosomal mRNA channel to inhibit translation. Nat Struct Mol Biol. 2020;27:959–66. 32908316 10.1038/s41594-020-0511-8

[18] Mendez AS , Ly M , Gonzalez‐Sanchez AM , Hartenian E , Ingolia NT , Cate JH , et al. The N‐terminal domain of SARS‐CoV‐2 nsp1 plays key roles in suppression of cellular gene expression and preservation of viral gene expression. Cell Rep. 2021;37:109841. 34624207 10.1016/j.celrep.2021.109841 PMC8481097

[19] Kumar A , Kumar A , Kumar P , Garg N , Giri R . SARS‐CoV‐2 NSP1 C‐terminal (residues 131–180) is an intrinsically disordered region in isolation. Curr Res Virol Sci. 2021;2:100007. 34189489 10.1016/j.crviro.2021.100007 PMC8020630

[20] Slavin M , Zamel J , Zohar K , Eliyahu T , Braitbard M , Brielle E , et al. Targeted in situ cross‐linking mass spectrometry and integrative modeling reveal the architectures of three proteins from SARS‐CoV‐2. Proc Natl Acad Sci USA. 2021;118:e2103554118. 34373319 10.1073/pnas.2103554118 PMC8403911

[21] Tidu A , Janvier A , Schaeffer L , Sosnowski P , Kuhn L , Hammann P , et al. The viral protein NSP1 acts as a ribosome gatekeeper for shutting down host translation and fostering SARS‐CoV‐2 translation. RNA. 2020;27:253–64. 10.1261/rna.078121.120 PMC7901841 33268501

[22] Tanaka T , Kamitani W , DeDiego ML , Enjuanes L , Matsuura Y . Severe acute respiratory syndrome coronavirus nsp1 facilitates efficient propagation in cells through a specific translational shutoff of host mRNA. J Virol. 2012;86:11128–37. 22855488 10.1128/JVI.01700-12 PMC3457165

[23] Loureiro CL , Jaspe RC , D'Angelo P , Zambrano JL , Rodriguez L , Alarcon V , et al. SARS‐CoV‐2 genetic diversity in Venezuela: predominance of D614G variants and analysis of one outbreak. PLoS One. 2021;16:e0247196. 33606828 10.1371/journal.pone.0247196 PMC7895374

[24] Mercatelli D , Giorgi FM . Geographic and genomic distribution of SARS‐CoV‐2 mutations. Front Microbiol. 2020;11:1800. 32793182 10.3389/fmicb.2020.01800 PMC7387429

[25] Lin JW , Tang C , Wei HC , Du B , Chen C , Wang M , et al. Genomic monitoring of SARS‐CoV‐2 uncovers an Nsp1 deletion variant that modulates type I interferon response. Cell Host Microbe. 2021;29:489–502.e8. 33548198 10.1016/j.chom.2021.01.015 PMC7846228

[26] Benedetti F , Snyder GA , Giovanetti M , Angeletti S , Gallo RC , Ciccozzi M , et al. Emerging of a SARS‐CoV‐2 viral strain with a deletion in nsp1. J Transl Med. 2020;18:329. 32867854 10.1186/s12967-020-02507-5 PMC7457216

[27] Clark LK , Green TJ , Petit CM . Structure of nonstructural protein 1 from SARS‐CoV‐2. J Virol. 2021;95:e02019‐20. 33234675 10.1128/JVI.02019-20 PMC7851544

[28] Almeida MS , Johnson MA , Herrmann T , Geralt M , Wuthrich K . Novel beta‐barrel fold in the nuclear magnetic resonance structure of the replicase nonstructural protein 1 from the severe acute respiratory syndrome coronavirus. J Virol. 2007;81:3151–61. 17202208 10.1128/JVI.01939-06 PMC1866046

[29] Gordon DE , Jang GM , Bouhaddou M , Xu J , Obernier K , White KM , et al. A SARS‐CoV‐2 protein interaction map reveals targets for drug repurposing. Nature. 2020;583:459–68. 32353859 10.1038/s41586-020-2286-9 PMC7431030

[30] Andreev DE , Dmitriev SE , Terenin IM , Prassolov VS , Merrick WC , Shatsky IN . Differential contribution of the m7G‐cap to the 5' end‐dependent translation initiation of mammalian mRNAs. Nucleic Acids Res. 2009;37:6135–47. 19696074 10.1093/nar/gkp665 PMC2764426

[31] Zeenko VV , Wang C , Majumder M , Komar AA , Snider MD , Merrick WC , et al. An efficient in vitro translation system from mammalian cells lacking the translational inhibition caused by eIF2 phosphorylation. RNA. 2008;14:593–602. 18230759 10.1261/rna.825008 PMC2248251

[32] Carroll R , Lucas‐Lenard J . Preparation of a cell‐free translation system with minimal loss of initiation factor eIF‐2/eIF‐2B activity. Anal Biochem. 1993;212:17–23. 8368492 10.1006/abio.1993.1284

[33] Takada H , Roghanian M , Murina V , Dzhygyr I , Murayama R , Akanuma G , et al. The C‐terminal RRM/ACT domain is crucial for fine‐tuning the activation of ‘long’ RelA‐SpoT homolog enzymes by ribosomal complexes. Front Microbiol. 2020;11:277. 32184768 10.3389/fmicb.2020.00277 PMC7058999

[34] Huynh K , Partch CL . Analysis of protein stability and ligand interactions by thermal shift assay. Curr Protoc Protein Sci. 2015;79:28.9.1–28.9.14. 25640896 10.1002/0471140864.ps2809s79 PMC4332540

[35] Jumper J , Evans R , Pritzel A , Green T , Figurnov M , Ronneberger O , et al. Highly accurate protein structure prediction with AlphaFold. Nature. 2021;596:583–9. 34265844 10.1038/s41586-021-03819-2 PMC8371605

[36] Eastman P , Swails J , Chodera JD , McGibbon RT , Zhao Y , Beauchamp KA , et al. OpenMM 7: rapid development of high performance algorithms for molecular dynamics. PLoS Comput Biol. 2017;13:e1005659. 28746339 10.1371/journal.pcbi.1005659 PMC5549999

[37] Goddard TD , Huang CC , Meng EC , Pettersen EF , Couch GS , Morris JH , et al. UCSF ChimeraX: meeting modern challenges in visualization and analysis. Protein Sci. 2018;27:14–25. 28710774 10.1002/pro.3235 PMC5734306

[38] Bond CS . TopDraw: a sketchpad for protein structure topology cartoons. Bioinformatics. 2003;19:311–2. 12538265 10.1093/bioinformatics/19.2.311

[39] Lo MC , Aulabaugh A , Jin G , Cowling R , Bard J , Malamas M , et al. Evaluation of fluorescence‐based thermal shift assays for hit identification in drug discovery. Anal Biochem. 2004;332:153–9. 15301960 10.1016/j.ab.2004.04.031

