# Supplementary figures and images for: Clinically observed deletions in SARS‐CoV‐2 Nsp1 affect its stability and ability to inhibit translation
Source: FEBS Lett. 2022 Apr 25;596(9):1203–13. doi: 10.1002/1873-3468.14354 (PMC9081967; doi:10.1002/1873-3468.14354)

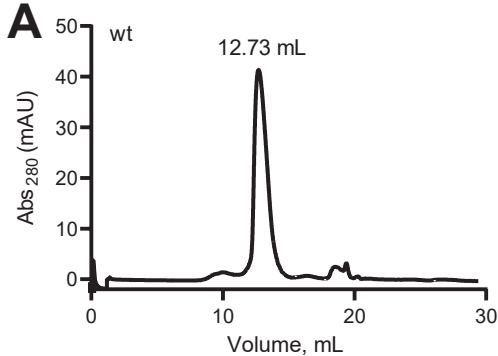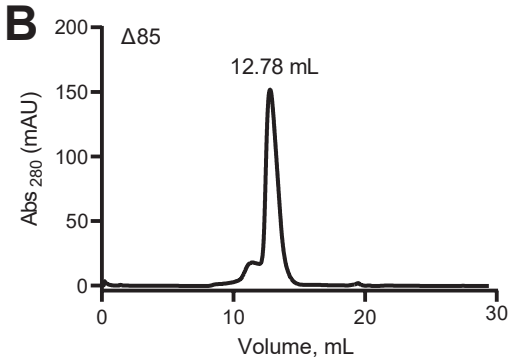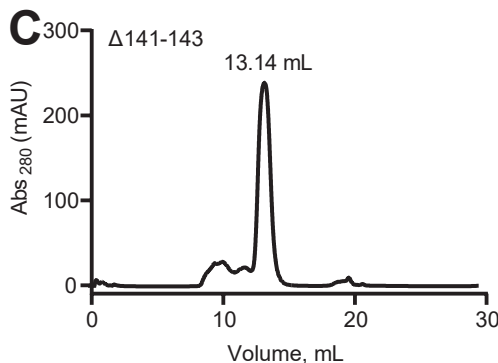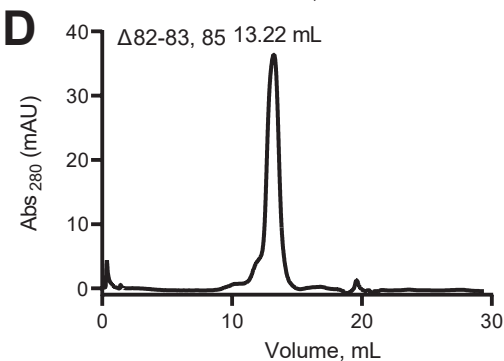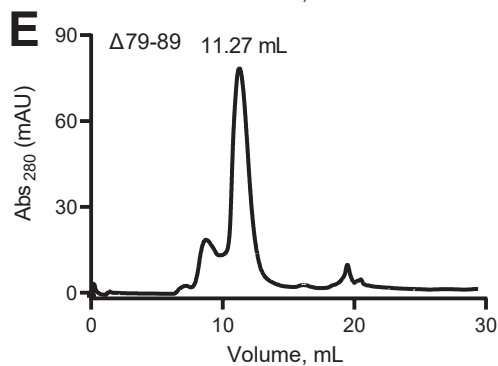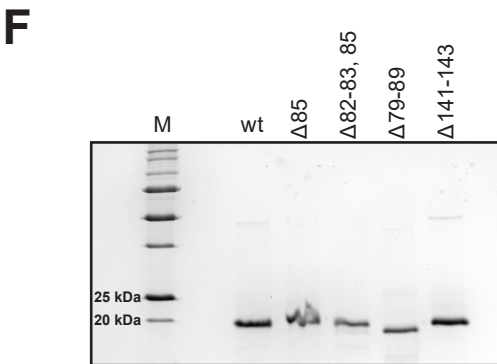

Supplement: Supplementary file 1 — Fig. S1. Purification of wild type and deletion variants of SARS‐CoV‐2 Nsp1. (A‐E) Chromatograms (280 nm absorption) of Nsp1 and its deletion variants eluting from a Superdex 75 increase 10/300 size‐exclusion column. Each chromatogram has the volume corresponding to the center of the main peak noted. The A260/A280 ratio was similar (~0.6) in the main peak of all constructs. (F) 1.5 μg of each protein sample was analyzed on a 15% SDS‐PAGE and stained with Coomassie blue. [file FEB2-596-1203-s002.pdf]

**A**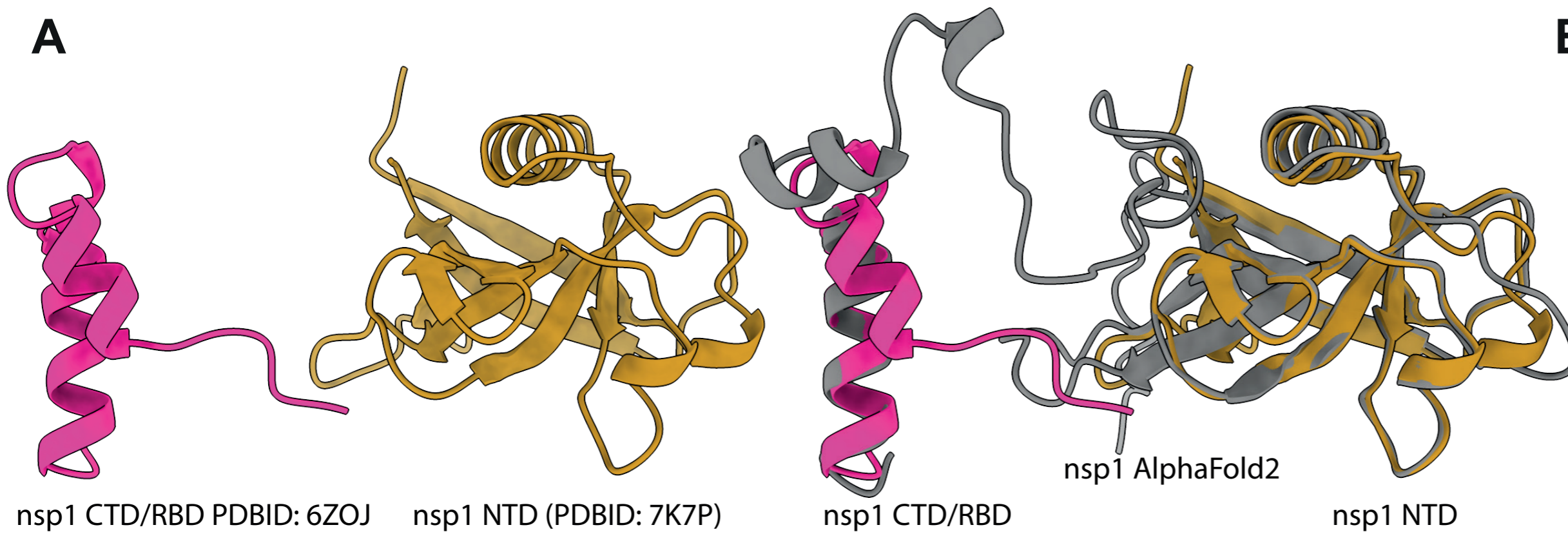**B**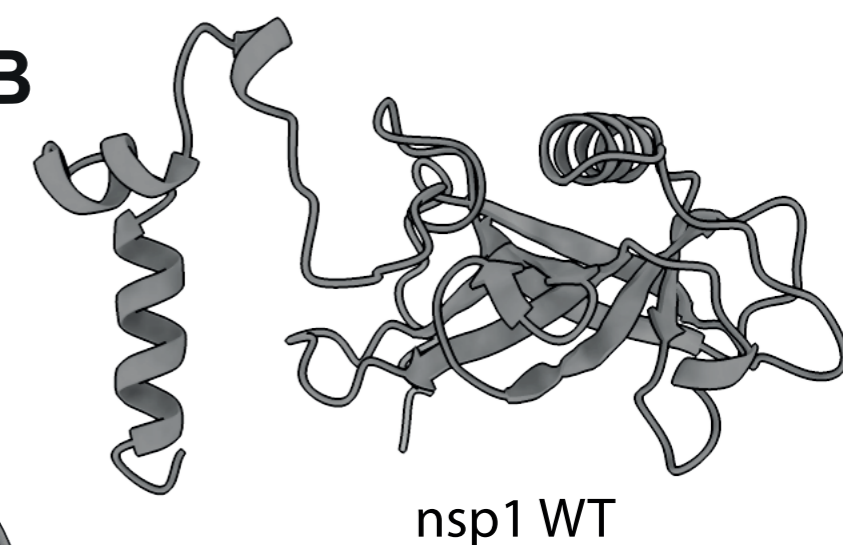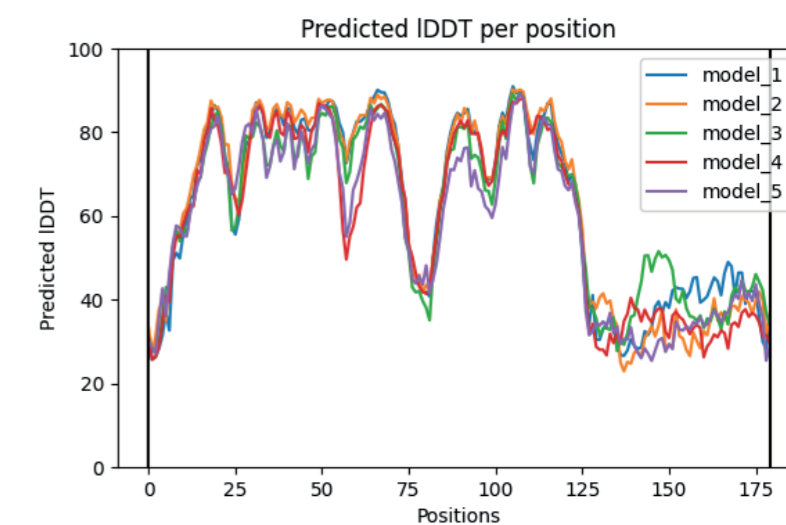**C**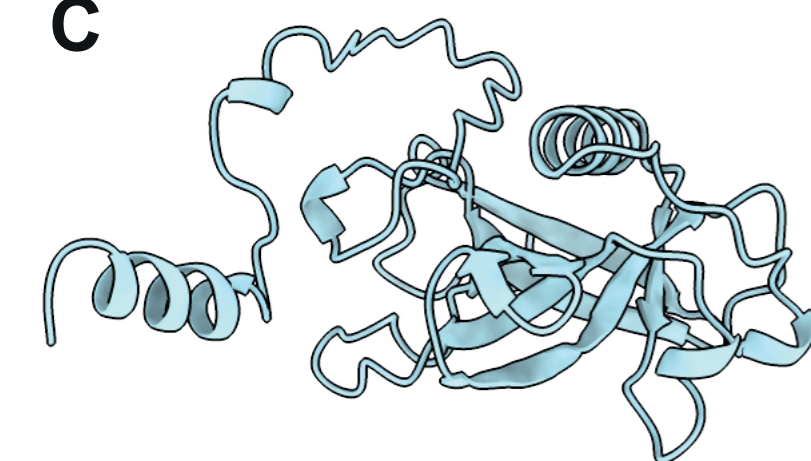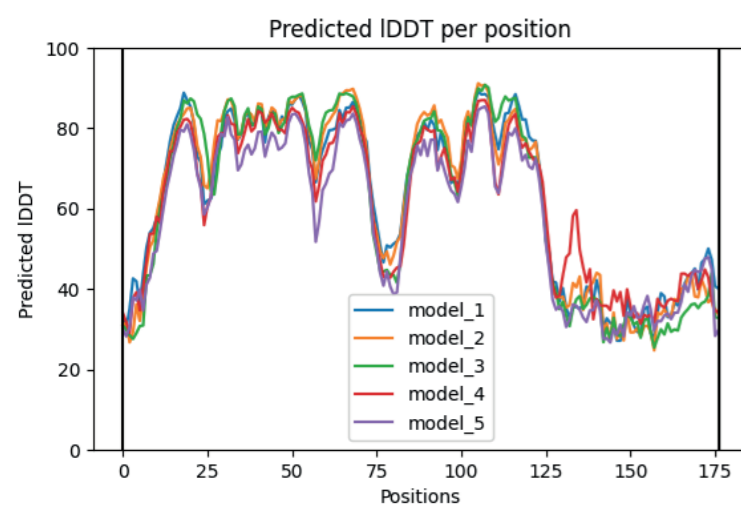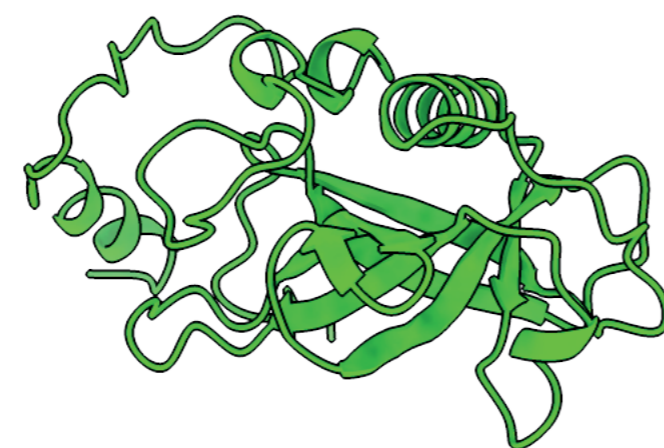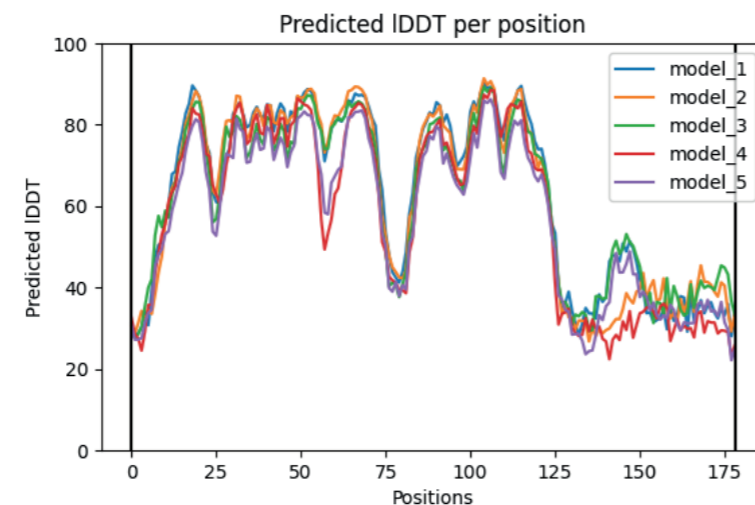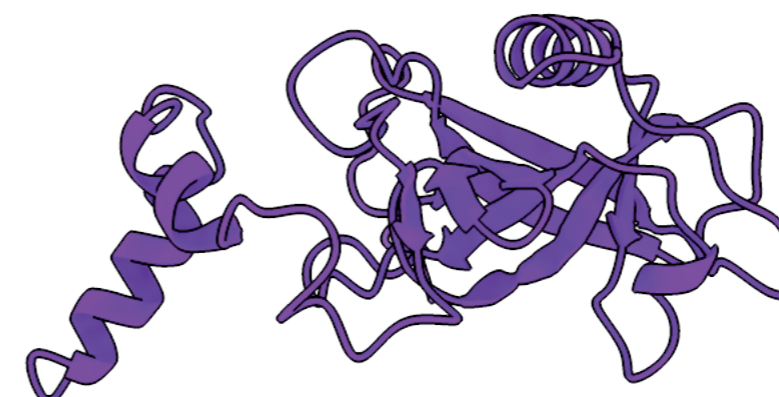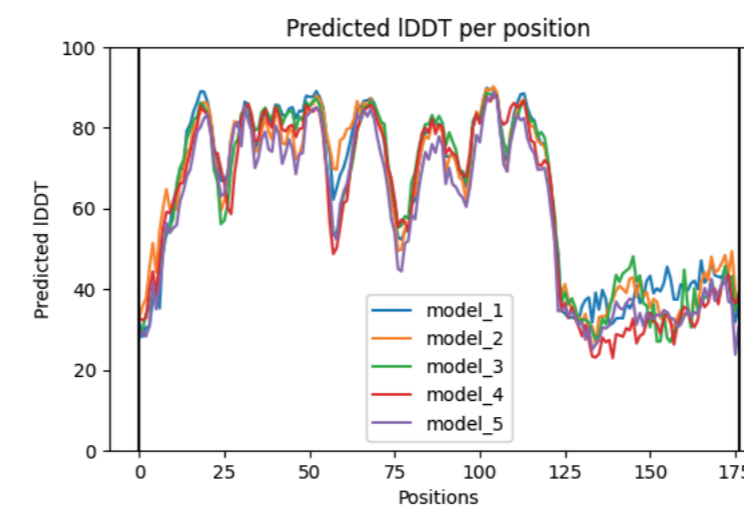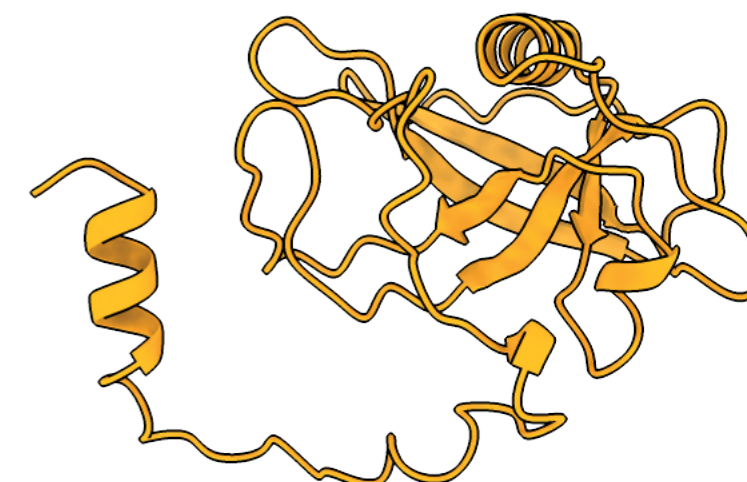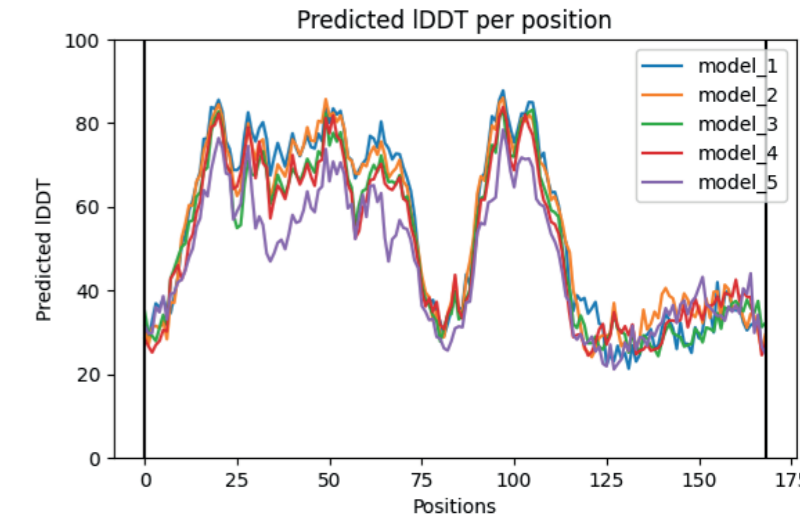

Supplement: Supplementary file 2 — Fig. S2. AlphaFold2‐predicted Nsp1 structures. (A) (left) Structures of the experimentally determined N‐ (residues 10‐126, PDBID: 7K7P[27]) and C‐terminal (residues 148‐180 PDBID: 6ZOJ[17]) domains of Nsp1. Structures are oriented as the predicted full‐length structure of wt Nsp1 in (B). (right) Superposition of the predicted wt Nsp1 structure with the experimentally determined structures as in (A). (B) Predicted structure of full length wt Nsp1 shown in cartoon representation (grey) and the corresponding per‐residue confidence score (pLDDT) trace for all five prediction runs. (C) Cartoon representations of the predicted structures of Δ141‐143 (light blue), Δ85 (green), Δ82‐83,85 (purple) and Δ79‐89 (orange) Nsp1 and their respective per‐residue confidence score (pLDDT) trace for each of their predictions. [file FEB2-596-1203-s001.pdf]
